# Parasite densities modulate susceptibility of mice to cerebral malaria during co-infection with *Schistosoma japonicum* and *Plasmodium berghei*

**DOI:** 10.1186/1475-2875-13-116

**Published:** 2014-03-26

**Authors:** Mei-lian Wang, Yong-hui Feng, Wei Pang, Zan-mei Qi, Ying Zhang, Ya-jun Guo, En-jie Luo, Ya-ming Cao

**Affiliations:** 1 Department of Microbiology and Parasitology, College of Basic Medical Sciences, China Medical University, No 92 Beier Road, Heping District, Shenyang 110001, China; 2 Department of Immunology, College of Basic Medical Sciences, China Medical University, No 92 Beier Road, Heping District, Shenyang 110001, China; 3 Department of Sonography, Shengjing Hospital of China Medical University, No 36 Sanhao Street, Heping District, Shenyang 110004, China

**Keywords:** *Plasmodium berghei* ANKA, *Schistosoma japonicum*, Co-infection, Inflammatory cytokine, Parasite density, Malaria

## Abstract

**Background:**

Malaria and schistosomiasis are endemic and co-exist in the same geographic areas, even co-infecting the same host. Previous studies have reported that concomitant infection with *Schistosoma japonicum* could offer protection against experimental cerebral malaria (ECM) in mice. This study was performed to evaluate whether alterations in parasite density could alter this protective effect.

**Methods:**

Mice were inoculated with 100 or 200 *S. japonicum* cercariae followed by infection with high or low density of *Plasmodium berghei* ANKA strain eight weeks after the first infection. Then, parasitaemia, survival rate and blood–brain-barrier (BBB) damage were assessed. Interferon-gamma (IFN-γ), interleukin (IL)-4, IL-5, IL-13, IL-10, and TGF-β levels were determined in splenocyte supernatants using enzyme-linked immunosorbent assay (ELISA). Cell surface/intracellular staining and flow cytometry were used to analyse the level of CD4^+^/CD8^+^ T cells, CD4^+^CD25^+^Foxp3^+^ Tregs, IL-10-secreting Tregs, and IL-10^+^Foxp3^-^CD4^+^ T cells in the spleen, and CD4^+^/CD8^+^ T cells infiltrating the brain.

**Results:**

Co-infection with low density *P. berghei* and increased *S. japonicum* cercariae significantly increased the levels of IL-4, IL-5, IL-13, TGF-β and Tregs, but significantly decreased the levels of IFN-γ and the percentage of CD4^+^ T cells and CD8^+^ T cells in the spleen and CD8^+^ T cell infiltration in the brain. Increased worm loads also significantly decreased mortality and BBB impairment during ECM. When challenged with higher numbers of *P. berghei* and increased cercariae, the observed cytokine changes were not statistically significant. The corresponding ECM mortality and BBB impairment also remained unchanged.

**Conclusions:**

This study demonstrates that protection for ECM depends on the numbers of the parasites, *S. japonicum* and *P. berghei*, during co-infection. Alterations in the regulatory response appear to play a key role in this adaptation.

## Background

Malaria, caused by the infection of parasites belonging to *Plasmodium* species, is a major health problem for humans in many tropical regions of the world. This devastating disease kills more than one million children each year [1]. Specifically, cerebral malaria (CM) is one of the most severe complications of *Plasmodium* infection and a major cause of death among young children in sub-Saharan Africa [2]. Precise mechanisms of CM onset are still incompletely understood as multiple factors including both host and pathogen determinants are involved in the pathogenesis [3]. Overall, it is considered that the sequestration of parasitized red blood cells (pRBCs) in cerebral microvasculature contributes to vessel occlusion, hypoxia, endothelial activation and blood–brain-barrier (BBB) dysfunction [4-6].

A number of studies have documented that CM results from a predominant Th1 response. Besides, a number of pro-inflammatory cytokines, including IFN-γ and relatively lower levels of anti-inflammatory cytokines, such as IL-10 were recorded in individuals with severe malaria [7,8]. It is generally considered that a proper balance between pro- and anti-inflammatory molecules is essential to control the pathogenesis of severe malaria [9]. While lack of an initial inflammatory stage may lead to increased parasite proliferation, an uncontrolled inflammatory response may lead to severe immunopathology [10]. In this molecular balance, CD4^+^CD25^+^Foxp3^+^ regulatory T cells (Tregs) play a critical role. A previous report demonstrated that Tregs are required to limit pro-inflammatory immune responses in BALB/c mice to prevent experimental cerebral malaria (ECM) during secondary infections [11]. It has also been confirmed that the occurrence of Tregs during *Plasmodium berghei* ANKA infection is negatively associated with the production of IFN-γ [12]. Induced and/or activated Tregs may be beneficial to the vertebrate host because it down-regulates the inflammatory response and thereby prevents immune-mediated pathology.

Since helminths and malarial parasites share the same geographical distribution, they are known to infect the same vertebrate host population [13]. Therefore, it is important to elucidate the immune mechanisms underlying the co-infection of these parasites. Both malaria and helminthiasis induce strong immune responses affecting the Th1/Th2 balance [13,14]. In a previous study, it was documented that pre-existing *Schistosoma japonicum* infection strengthened the Tregs-associated Th2 response to malaria infection and this Th2 response played an important role in protecting against ECM pathology [15]. The current study was designed to evaluate whether parasite density during co-infections modulated this protective effect that may reveal a parasite threshold for causing ECM.

## Methods

All experimental protocols in the current study were reviewed and approved by the Medical University Institute of Medical Research Animal Ethics Committee in China.

### Mice, parasites and experimental infection

Female C57BL/6 mice (four weeks old) were purchased from Beijing Animal Institute (Beijing, China) and maintained in individual ventilated cages in the animal facility at China Medical University. Mice were provided heat-sterilized food and distilled water *ad libitum*. C57BL/6 mice were selected as models in the current study because they showed high susceptibility to ECM due to their pro-inflammatory/Th1 predisposition.

The *S. japonicum* strain was obtained from Jiangsu Institute of Parasitic Diseases (Wuxi, China). *Plasmodium berghei* ANKA strain (clone 1.49 L) was provided by Dr. Motomi Torii at the Department of Molecular Parasitology, Ehime University Graduate School of Medicine, Ehime, Japan. Parasite stabilates were stored at -80°C. To obtain experimental inoculum of *P. berghei*, parasitized red blood cells (pRBCs) were sequentially passed through three homologous donor mice.

Infections were initiated in mice that were five weeks old. First, mice were infected percutaneously with 100 or 200 *S. japonicum* cercariae and eight weeks later mice in both groups were infected with higher dose (1 × 10^6^) or lower dose (1 × 10^5^) of *P. berghei* pRBCs. Control mice were infected with only *P. berghei* pRBCs or *S. japonicum* cercariae. Each group had 40 mice. The animals were euthanized one, three, five and eight days post-*P. berghei* infection for evaluation.

### Confirmation of helminth infection

Helminth infection was confirmed by the presence of worms and liver granulomas upon necropsy. Worms were obtained by portal perfusion as described previously [16], and livers were examined for the presence of granulomas under a stereomicroscope.

### Malaria parasitaemia, survival rates and disease assessment

Parasitaemia was determined by staining thin blood smears from mice tails with Giemsa and observation under light microscopy. Slides were coded and pRBCs were counted microscopically in at least five microscopic fields, each containing approximately 300 cells.

Every day post*-P. berghei* infection, mice were monitored for mortality*.* Clinical ECM was also assessed and was confirmed if at least four of the following signs were observed [12]: ruffled fur, hunching, wobbly gait, limb paralysis, convulsions, or coma. Each sign was given a score of 1. Animals with scores ≥4 were considered to have severe ECM.

### Spleen cell culture

Spleen cell culture was prepared as previously described [12,17]. Briefly, spleens from infected mice were aseptically removed and pressed individually through a sterile fine-wire mesh along with 10 ml RPMI-1640 (Life Technologies, Shanghai, China) supplemented with 5% heat-inactivated fetal calf serum (FCS; Hyclone Laboratories, Inc., South Logan, Utah, USA), 25 mM Hepes (Life Technologies), 0.12% gentamicin (Schering-Plough, Kenny Worth, New Jersey, USA), and 2 mM glutamine (Life Technologies). Cell suspensions were centrifuged at 350 g for 10 min at room temperature (RT). Erythrocytes were lysed in cold 0.17 M NH_4_Cl and the cells were washed twice in fresh medium. Viability of the spleen cells was determined by trypan blue exclusion assay, which revealed >90% cell survival. Spleen cells were adjusted to a final concentration of 10^7^ cells/ml in RPMI-1640 supplemented with 10% heat-inactivated FCS. Aliquots (500 μl/well) of the cell suspension were incubated in 24-well flat bottom culture plates (Falcon®, Corning Life Sciences, CA, USA) in triplicate for 48 hrs at 37°C in a humidified incubator with 5% CO_2_. The plates were then centrifuged at 350 g for 10 min at RT and the supernatants were collected and stored at -80°C until assayed for cytokine levels.

### Cytokine analysis

The levels of cytokines such as IFN-γ, IL-4, IL-5, IL-13, IL-10, and TGF-β in the splenocyte supernatants from infected mice were measured using commercial enzyme-linked immunosorbent assay (ELISA) kits according to the manufacturer’s instructions (R&D Systems, Minneapolis, MN, USA). All assays were performed in a microtitre plate reader and the absorbance was recorded at 450 nm. Cytokine concentrations in samples were calculated based on standard curves generated using recombinant cytokines.

### Cell surface/intracytoplasmic staining and flow cytometry

From each experimental group, five or six mice were sacrificed at the indicated time points for flow cytometry. Spleen cells were collected from C57BL/6 mice post infection (pi). Cell concentration was then adjusted to 2 × 10^6^/ml, followed by stimulation with plate-bound anti-mouse CD3 (1 μg/ml) and anti-mouse CD28 (0.2 μg/ml) together with Golgi Stop (BD Bioscience, Cat no 554724) for 4 hrs. After continued co-culture at 37°C for 4 hrs, cells were washed with 3% FCS and resuspended in 100 μl of 3% FCS followed by incubation with APC-conjugated anti-mouse-CD3, FITC-conjugated anti-mouse CD4, PerCP-Cy5.5-conjugated anti-mouse CD8 or PerCP-Cy5.5-conjugated anti-mouse CD25 (clone 3C7) for surface staining. The cells were then fixed, permeabilized and intracytoplasmic staining was performed using APC-conjugated anti-Foxp3 (clone FJK16s) or PE-conjugated anti-IL-10 (clone JES5-16E3). FITC-conjugated rat IgG2b was used as the isotype control.

To determine the migration of CD4^+^ and CD8^+^ T cells into the brain, mononuclear cells were isolated from the brains on day 6 pi. A single-cell suspension was obtained by homogenizing the tissues in 5 ml of RPMI 1640 containing 100 U/ml IV collagenase (Invitrogen, USA), and incubating at 42°C for 45 min. Cells were centrifuged at 300 g for 10 min, resuspended in 30% Percoll in PBS (Sigma), layered on 70% Percoll, and centrifuged at 515 g for 30 min at RT. Cells at the interface were isolated, washed twice, resuspended in PBS, and labeled with the following antibodies: FITC-anti-CD4, PerCP-Cy5.5-anti-CD8 (clone 53–6.7), or APC-anti-CD3 (clone 145-2C11) by incubating at 4°C for 30 min. After washing twice in PBS, flow cytometric analysis was performed using a FACS Calibur, and data were analysed with the FlowJo software.

### Assessment of BBB impairment

A 2% Evans blue dye was prepared with Evans blue powder (Sigma-Aldrich, St Louis, USA) and PBS, followed by filtration and sterilization. Mice were injected intravenously with 200 μl of 2% Evans blue on day 6 pi. The animals were then sacrificed 1 hr later and brains were isolated to evaluate vascular leakage of the dye into the brain parenchyma. After image documentation, brain tissues were weighed and placed in formamide for 48 hrs at 37°C to extract Evans blue. Aliquots (100 μl) of this solution were analysed on a microtitre plate. Absorbance was measured at 620 nm on an ELISA reader and compared to a standard curve [3]. Concentration of Evans blue was expressed as μg Evans blue per gram brain tissue.

### Statistical analysis

Data were analysed using Prism (GraphPad, La Jolla, CA, USA). Differences in survival was analysed using a Log-rank (Mantel-Cox) Test. Differences between multiple means were compared by one-way ANOVA, Tamhane’s T2 test or Bonferroni test when the variance was heterogeneous or homogeneous, respectively. All tests were considered significant when *P* < 0.05.

## Results

### *Schistosoma japonicum-*mono-infection

**Figure 1:**
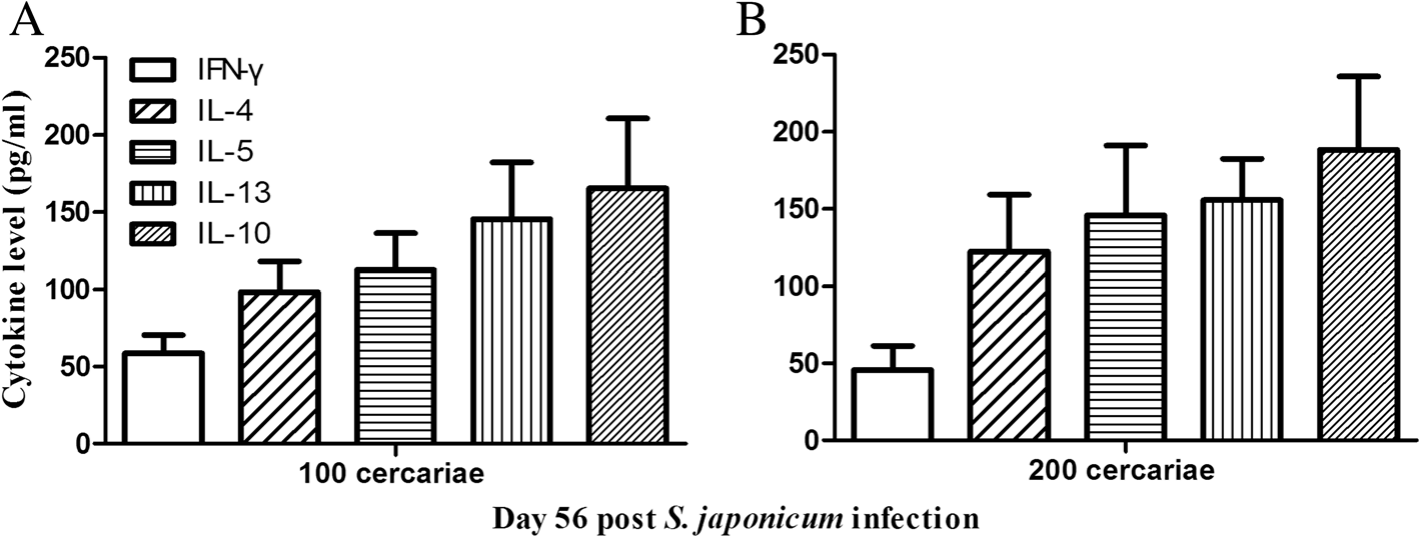
**Levels of splenic IFN-γ, IL-4, IL-5, IL-13 and IL-10 in C57BL/6 mice infected with 100 (A) or 200 (B) *S. japonicum *cercariae.** Splenocytes were isolated from mice on day 56 post*-S. japonicum* infection and cultured to measure indicated cytokine levels using ELISA. Bars represent mean values ± SD. n = 5-6 mice per group. Results are representative of three independent experiments.

All control mice infected with only 100 or 200 *S. japonicum* cercariae survived until day 75 post infection (> 10 weeks) after which they were sacrificed. Necropsy was performed and the results confirmed the existence of helminth infection in both groups. The levels of associated cytokines on day 56 (8 weeks) post infection indicated high Th1/Th2 levels just before the secondary infection with *P. berghei* (Figure 1). These results indicated that *S. japonicum* infection was in prime when secondary *P. berghei* infections were experimentally induced on week 8 post *S. japonicum* infection. In addition, previous studies [18] confirmed that responses to *Schistosoma* infections were highly Th2 polarized following the onset of egg production around week 6. In the current study, *S. japonicum* infection on week 8 was primed for a Th2 response.

### *Plasmodium berghei- Schistosoma japonicum*-co-infection

To evaluate the impact of parasite density in offering protection against the onset of ECM during co-infection, two schemes were included with two different densities of both parasites, *S. japonicum* and *P. berghei*. Because mice in the *S. japonicum-*mono-infected group received no additional treatment during the co-infection period, no changes were observed in the levels of the cytokines (data not shown).

#### Parasitaemia and survival rate

**Figure 2:**
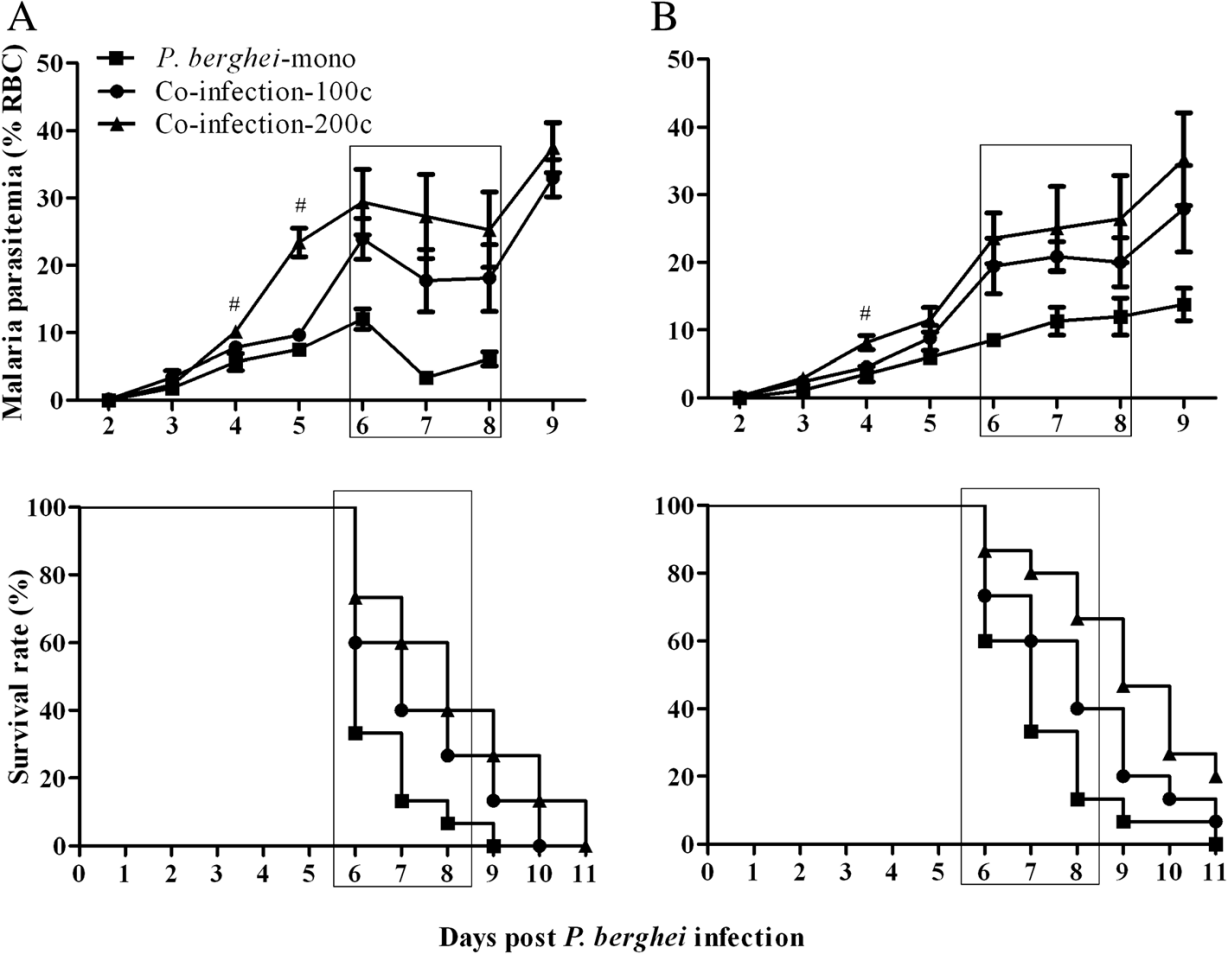
**Parasitaemia and survival rate of C57BL/6 mice co-infected with 100 or 200** ***S. japonicum *cercariae and 1 × 10**^**6 **^**(A) or 1 × 10**^**5 **^**(B) *P. berghei *pRBCs after eight weeks.** Intensities of *S. japonicum* cercariae in each panel are indicated as co-infection-200c and co-infection-100c, respectively. Mice infected with only *P. berghei* are indicated as *P. berghei*-mono. Malaria parasitaemia was monitored every day by staining blood samples from infected mice with Giemsa. Data are represented as mean ± SD. Rectangle boxes indicate periods susceptible to ECM. Significant differences between the groups in parasitaemia were determined by one-way ANOVA and survival was analysed using a Log-rank (Mantel-Cox) Test. ^#^indicates *P* < 0.01. n = 5–6 mice per group. Results are representative of three independent experiments.

Malaria parasitaemia and mortality were monitored in mice in the co-infection-100c and co-infection-200c groups, and challenged with 1 × 10^6^ *P. berghei* (Figure 2A) or by 1 × 10^5^ *P. berghei* (Figure 2B). In general, all mice showed increasing parasitaemia during the course of *P. berghei* infection. Among the groups, increasing worm loads resulted in a higher level of parasitaemia on day 4 and 5 pi, or day 4 pi when challenged with higher or lower density of *P. berghei*, respectively. Between days 6 and 8, which is the period of susceptibility to ECM, most mice presented clinical signs of ECM and subsequently died. Mice in the co-infection-200c group showed a significantly higher survival rate than the co-infection-100c mice group, when challenged with low density of *P. berghei*. However, the difference was not significant when co-infected with a higher level of *P. berghei*.

#### Cytokine concentrations

**Figure 3:**
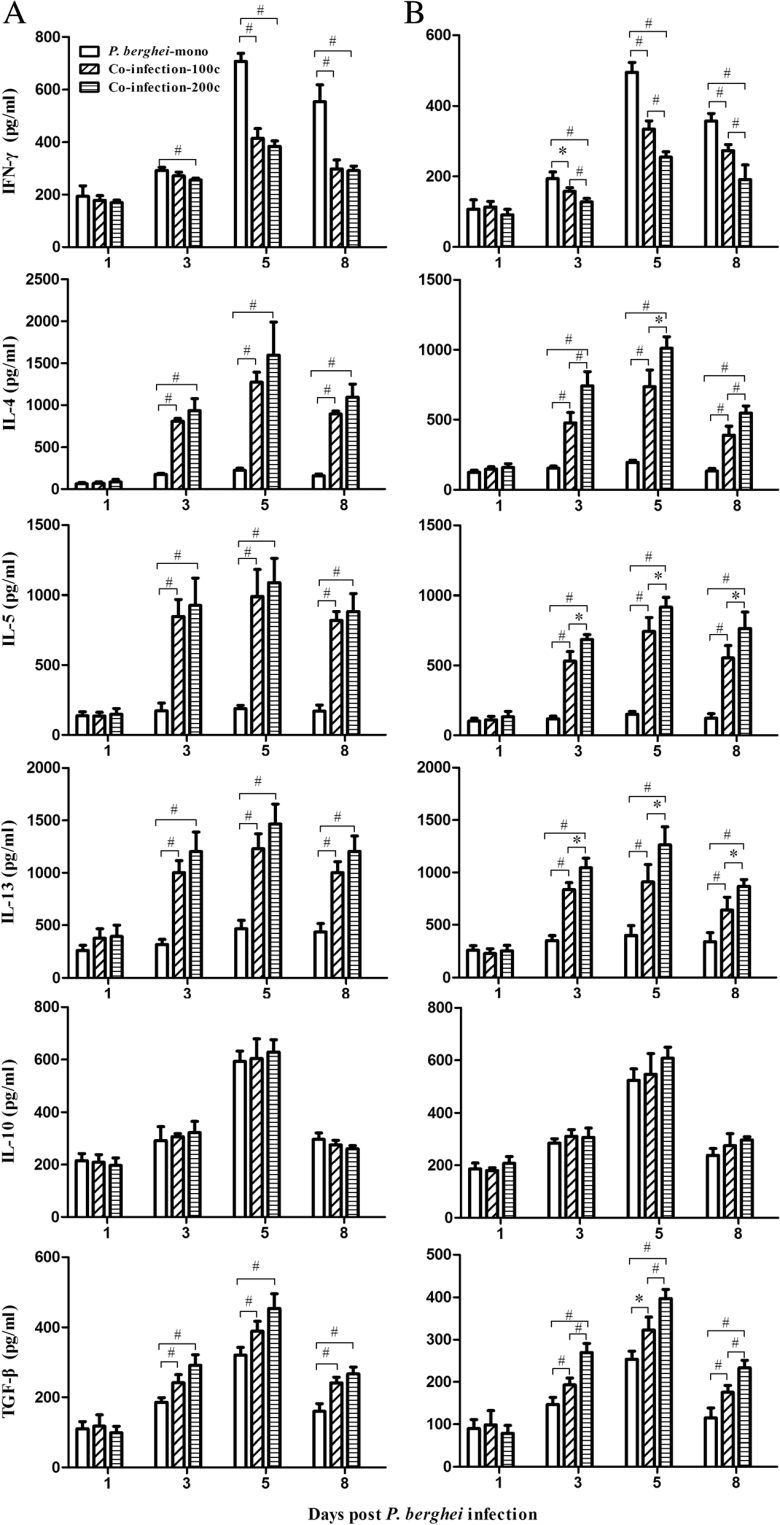
**Levels of splenic IFN-γ, IL-4, IL-5, IL-13, IL-10, and TGF-β in C57BL/6 mice co-infected with 100 or 200** ***S. japonicum *cercariae and 1 × 10**^**6 **^**(A) or 1 × 10**^**5 **^**(B) *P.berghei *pRBCs after eight weeks.** Intensities of *S. japonicum* cercariae in each panel are indicated as co-infection-200c and co-infection-100c, respectively. Mice infected with only *P. berghei* are indicated as *P. berghei*-mono. Splenocytes were isolated from mice on days 1, 3, 5, 8 post*-P. berghei* infection and cultured to measure indicated cytokine levels using ELISA. Bars represent mean values ± SD. Significant differences in cytokine levels over time and between indicated groups were determined by one-way ANOVA with *indicating *P* < 0.05 and ^#^indicating *P* < 0.01. n = 5-6 mice per group. Results are representative of three independent experiments.

To evaluate the relationship between the levels of pro- (IFN-γ) and anti-inflammatory (IL-4, IL-5, and IL-13) cytokines, and to assess cytokines involved in the regulatory responses (IL-10 and TGF-β) in the co-infected and *P. berghei*-mono-infected mice, cytokine levels in the supernatants of cultured splenocytes from mice in all groups were measured by ELISA. When infected with higher levels of *P. berghei* all cytokines including IFN-γ, IL-4, IL-5, IL-13, IL-10, and TGF-β increased immediately post-*P. berghei* infection, peaking on day 5 pi and then declining on day 8 pi (Figure 3A). Compared to *P. berghei*-mono, mice in the co-infection-100c group had significantly lower levels of IFN-γ on days 5 and 8 pi and higher levels of IL-4, IL-5, IL-13 and TGF-β on days 3, 5, and 8 pi, while no significant changes in IL-10 levels were observed between the two groups. A comparison between the co-infection-200c and co-infection-100c mice showed no significant difference among all pro- and anti-inflammatory cytokines measured. When challenged with lower density of *P. berghei*, the changes in cytokine levels over time were similar to those infected with high density of *P. berghei* (Figure 3B). Besides, co-infected mice showed significantly lower levels of IFN-γ and higher levels of IL-4, IL-5, IL-13, and TGF-β when compared to mice in *P. berghei*-mono group. Between the co-infection-200c and co-infection-100c groups, these cytokine changes were significant.

**Figure 4:**
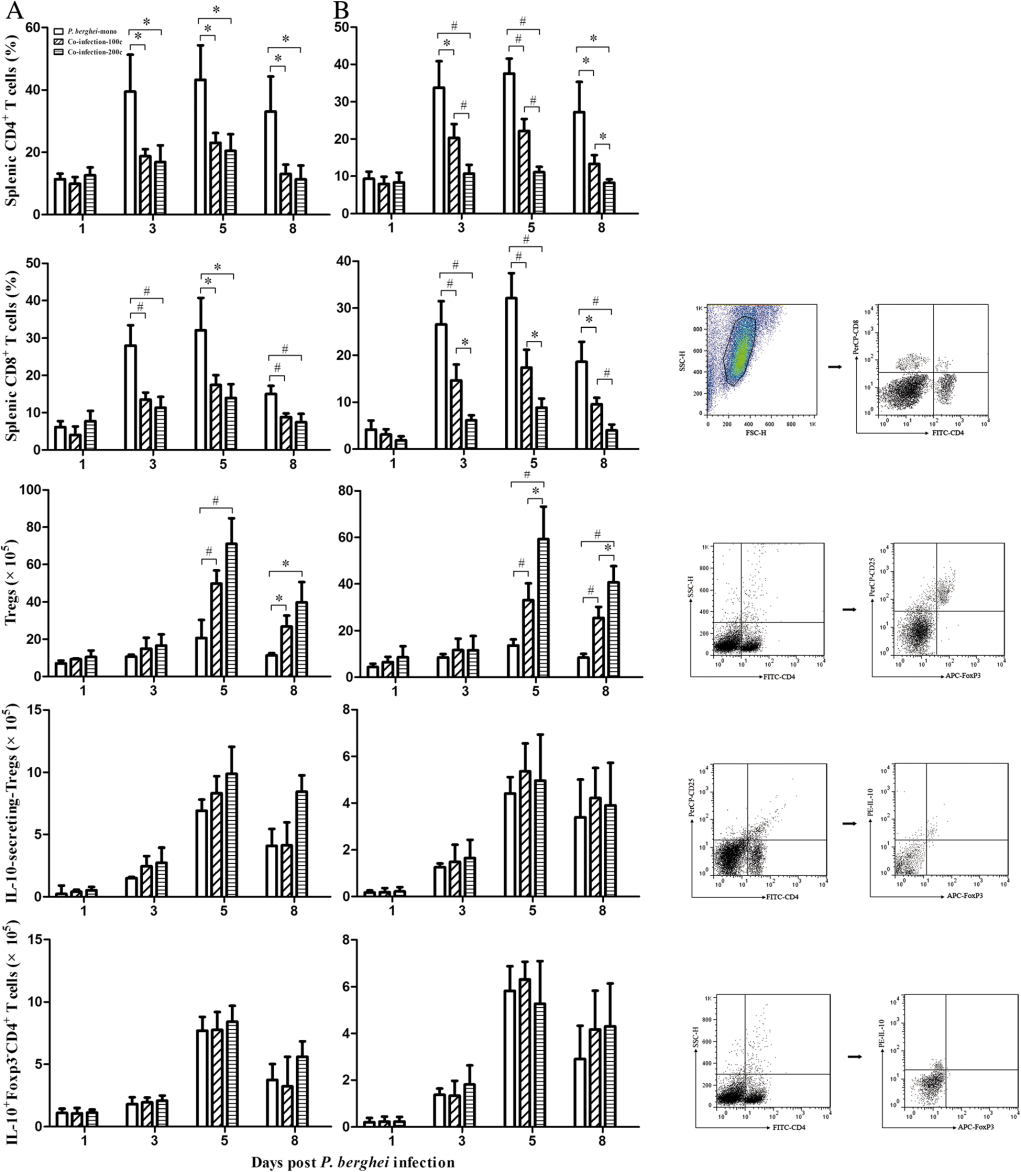
**The percentage of splenic CD4**^**+**^**/CD8**^**+ **^**T cells, and the absolutely numbers of CD4**^**+**^**CD25**^**+**^**Foxp3**^**+**^**Tregs, IL-10-secreting Tregs and IL-10**^**+**^**Foxp3**^**-**^**CD4**^**+ **^**T cells in C57BL/6 mice co-infected with 100 or 200** ***S. japonicum *cercariae and 1 × 10**^**6 **^**(A) or 1 × 10**^**5 **^**(B) *P. berghei *pRBCs eight weeks later.** Intensities of *S. japonicum* cercariae in each panel are indicated as co-infection-200c and co-infection-100c, respectively. Mice infected with only *P. berghei* are indicated as *P. berghei*-mono. Mice were dissected on days 1, 3, 5, 8 post*-P. berghei* infection to obtain the respective cell counts. Bars represent the mean values ± SD. One-way ANOVA was performed to compare values between indicated groups with *indicating *P* < 0.05 and ^#^indicating *P* < 0.01. n = 5–6 mice per group. FACS plots were used to show the cell populations and the gating strategy.

The kinetics of T cells population assessed by flow cytometry revealed that both splenic CD4^+^ and CD8^+^ T cells expanded during the course of *P. berghei* infection (Figure 4). In the two different *S. japonica* schemes, mice in the co-infection-100c group had significantly lower levels of CD4^+^ and CD8^+^ T cells on days 3, 5 and 8 pi when compared to *P. berghei*-mono group. Interestingly, these differences were observed only when challenged with lower density of *P. berghei* when made comparisons between the co-infection-200c and co-infection-100c groups. The kinetics of Tregs in both experimental schemes was also evaluated (Figure 4). Both IL-10-secreting Tregs, and IL-10 producing Foxp3^-^CD4^+^ T cells increased when challenged with higher (Figure 4A) or lower (Figure 4B) levels of *P. berghei*, peaking on day 5 pi and declining on day 8 pi. Among the two experimental schemes, the co-infection-100c group showed significantly higher levels of Tregs on days 5 and 8 pi when compared to *P. berghei*-mono group. Between the co-infection-200c and co-infection-100c groups, a significant change in this cytokine was present only when challenged with lower levels of *P. berghei*. In both experiments, no significant changes were observed in IL-10-secreting-Tregs and IL-10^+^Foxp3^-^CD4^+^ T cells.

**Figure 5:**
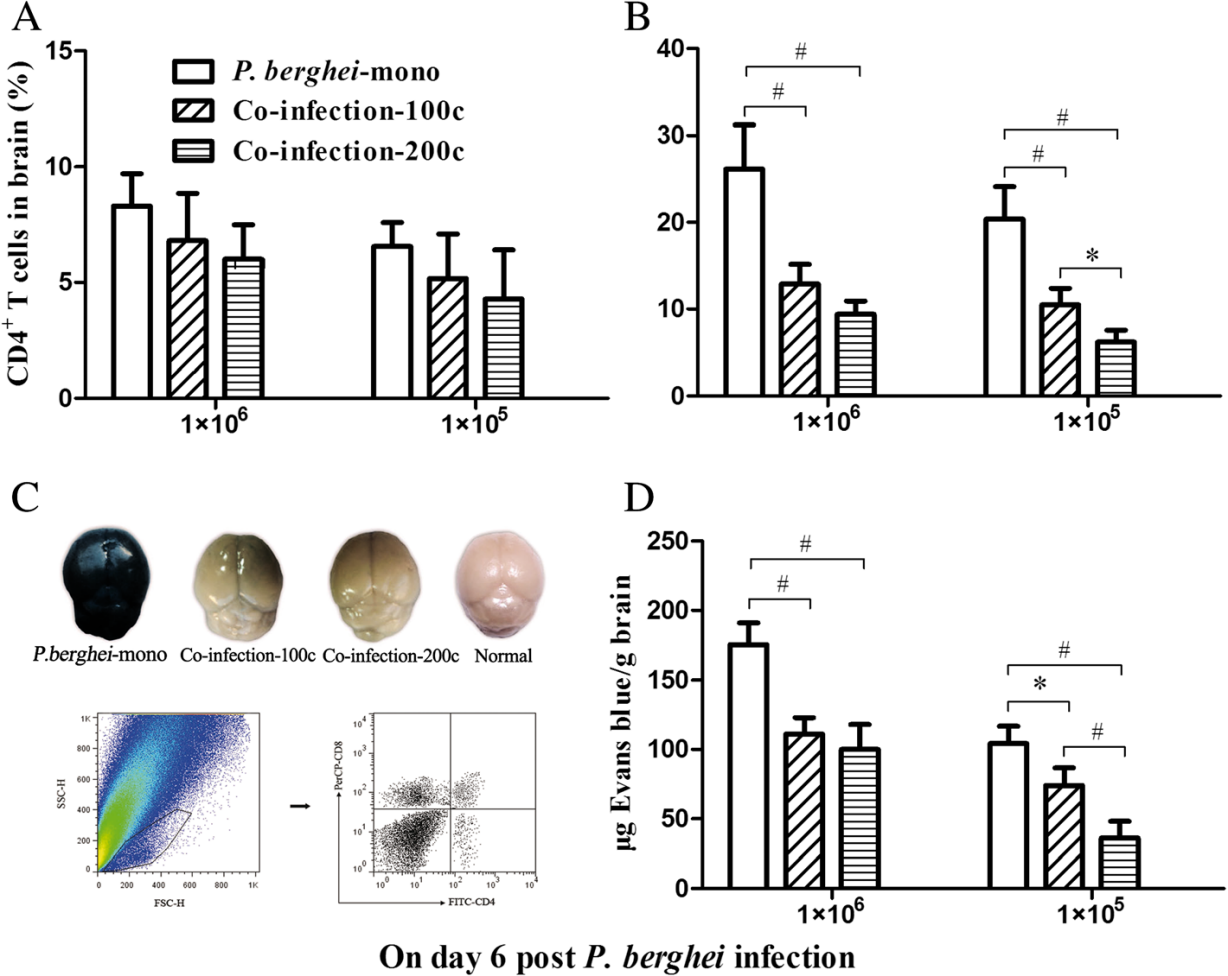
**The percentage of CD4**^**+ **^**(A) and CD8**^**+ **^**(B) T cells in the brain of C57BL/6 mice, which were co-infected with 100 or 200** ***S. japonicum *cercariae and 1 × 10**^**6 **^**or 1 × 10**^**5 **^***P. berghei *pRBCs eight weeks later.** Intensities of *S. japonicum* cercariae in each panel are indicated as co-infection-200c and co-infection-100c, respectively. Mice infected with only *P. berghei* are indicated as *P. berghei*-mono. Mice were dissected on days 6 post*-P. berghei* infection to obtain the respective cell counts. In addition, BBB damage was evaluated by the blue discoloration of the Evans blue stained brain tissues. Representative examples for *P. berghei*-mono-infection, co-infection-100c, co-infection-200c, and normal controls are shown in panel **(C)**. Quantification of Evans blue extravasation in formamide was measured as absorbance at 620 nm **(D)**. Bars represent the mean values ± SD. One-way ANOVA was performed to compare values between indicated groups with *indicating *P* < 0.05 and ^#^indicating *P* < 0.01. n = 5–6 mice per group. FACS plots were used to show the cell populations and the gating strategy.

To determine whether parasite dosage affected the migration and accumulation of T cells in the brains of *S. japonicum*-*P. berghei*-co-infected mice, mononuclear cells were isolated from the brain followed by quantification of CD4^+^ (Figure 5A)and CD8^+^ (Figure 5B) T cells. Compared to *P. berghei-*mono-infected mice, those in the co-infection-100c group recorded a significantly lower CD8^+^ T cell accumulation when challenged with high or low density of *P. berghei*. In contrast, when compared between co-infection-200c and co-infection-100c mice, such significant change was present only when challenged with low density of *P. berghei*.

#### BBB damage assessment

One of the hallmarks of CM is the damage of BBB integrity, which was analysed in all experiments both qualitatively and quantitatively. When compared to *P. berghei-*mono-infected mice, those co-infected with higher or lower densities of *P. berghei* along with *S. japonicum* displayed weak staining. A representative result in Figure 5C shows brains of mice challenged with 1 × 10^6^*P. berghei* ranging in color from dark blue, moderate blue, faint blue and unstained brain in *P. berghei*-mono, co-infection-100c, co-infection-200c, and normal control mice, respectively. In addition, colorimetric analysis of co-infected mice also showed significantly lower mass ratio of Evans blue/brain tissue than *P. berghei-*mono-infected mice. However, a comparison between co-infection-200c and co-infection-100c mice revealed that the significance was present only in those challenged with low density of *P. berghei* (Figure 5D).

## Discussion

Epidemiological studies on malaria and schistosomiasis suggest common immune responses to co-infections in people living in areas where these diseases are co-endemic [19]. A previous study documented the existence of shared antigens in the pathogens and cross-reactive antibodies to different components of the two parasites [20]. It is well known that *Schistosoma* infections have potent immunomodulatory effects on their hosts based on their anti-inflammatory effect, which dampens IFN-γ responses and switches immune responses toward a Th2 type response through Tregs [10]. During the past decade, many studies examined the impact of malaria co-infection with schistosomiasis although the results were contradicting. In a *Plasmodium yoelii* murine model, co-existing *Schistosoma mansoni* infection appeared to increase parasitaemia and the lethal disease [21]. Waknine-Grinberg *et al.*[22] and Bucher *et al.*[9] demonstrated that *S. mansoni* co-infected with *P. berghei* offered protection against cerebral malaria, while Lyke *et al.*[23] and Thigpen *et al.*[24] showed that *Schistosoma haematobium* infection decreased Tregs and *Plasmodium* parasitaemia, combined with increased IFN-γ and IL-2 during malaria infection. Thus, *S. haematobium* infection appears to increase the inflammatory response following malaria infection. Moreover, a study in Thailand documented that *Plasmodium falciparum* malaria increased in helminth-infected patients but reduced cerebral malaria and acute renal failure [25]. These divergent results may be caused by the use of different *Plasmodium* strains co-infected with different *Schistosoma* strains*,* each in various quantities with different but overlapping influences on immunity [25].

Currently, *S. mansoni* is the most widely used *Schistosoma* species to evaluate host immune responses [26] and only a few studies have used *S. japonicum* to create a *Schistosoma*-*Plasmodium*-co-infection murine model. A previous study demonstrated that *S. japonicum* co-infection could offer protection against cerebral malaria by enhancing Th2 responses [15]. The current study further examined the interactions between *S. japonicum* and *P. berghei* when the worm loads and *Plasmodium* densities were changed by evaluating several critical factors, such as malaria parasitaemia, Th1/Th2 responses, and the regulatory responses.

*Plasmodium* parasitaemia could be affected by multiple factors, including the dose of parasites infecting a host, immunity status of the host, and additional antigen interference, such as concomitant infection with *Schistosoma*. In general, it is believed that concomitant infection with *Schistosoma* could increase *Plasmodium* parasitaemia, which is consistent with results from the current study. This study also indicated that increasing the cercariae load also increased the parasitaemia in co-infected mice. The likely cause for this is the enhanced Th2 response when increasing the worm load. The associated Th1/Th2 cytokine analysis in future investigations will offer additional insights into the regulation of this mechanism.

Previous reports [22] have indicated the role of cytokines IFN-γ, IL-4, IL-5, and IL-13 in response to co-infections with *Plasmodium* and *Schistosoma.* Wang *et al.* also documented an increase in Th2 cytokines and decline in Th1 cytokines concomitant with *S. japonicum* infection [15]. Existing evidence suggests that increased worm load could lead to a more polarized Th2 response during *Schistosoma-Plasmodium-*co-infection. Yet this occurred only when the density of *Plasmodium* was low suggesting that the previous speculation of the immunomodulatory mechanism may be an oversimplification and may not apply at least for co-infections with worms and malaria parasites. Immune response during co-infections is complex largely due to an interactive effect between the responses triggered by two parasites. *Schistosoma* infection could down-regulate Th1 responses induced by *Plasmodium* infection although this effect may be suppressed when *Plasmodium*-induced Th1 response is too strong, as evidence in this study in mice challenged with high density of *P. berghei*. Taken together, these findings indicate that increased worm loads may lead to a decrease in Th1 response with concomitant *Plasmodium* infection, while the reduction magnitude may depend on *Plasmodium* density.

Another hypothesis in the present study is that regulatory responses, including Tregs, IL-10 and TGF-β, play a role in the immune responses of the vertebrate host during co-infections. Previous studies have reported the involvement of Tregs in the immune responses to both malaria [11,12] and schistosomiasis [27] infections. *In vitro* culture experiments have suggested that IL-10 and TGF-β are major cytokines that drive the induction and expansion of Tregs [28]. Results of the current study indicate that TGF-β plays a major role in the induction of Tregs mediated anti-inflammatory responses as evidenced by a significant increase in TGF-β levels in co-infected mice while the IL-10 levels remained unchanged. In general, Tregs is known to play two major roles in host immunity. First, Tregs could block T cell-mediated clearance of malaria parasites, facilitating an increase in parasitaemia. Second, Tregs could potentially play a beneficial role in preventing immunopathology during the infection [29]. In a previous study [29], Ashraful *et al*. documented a Tregs-induced reduction in T cell-mediated parasite tissue sequestration in a mouse malaria model, thereby limiting malaria-induced immune pathology. The study reported that total depletion of natural Tregs could not offer protection against ECM. In contrast, sufficient expansion of Tregs via IL-2Jc during *P. berghei* infection could suppress normal pathogenic T cell responses and prevented ECM. Pre-existing *S. japonicum* infection has been also reported to increase the absolute number of Tregs in splenocytes [15]. The findings of the current study corroborate these data and further extend the occurrence of increased Tregs numbers to increased worm loads during the co-infection with lower density *P. berghei*. This immune response could indeed offer more protection against ECM.

Based on previous pathophysiological manifestations, C57BL/6 mice animal models were highly susceptible to *P. berghei* infections. In this study, BBB impairment was chosen for analysis rather than brain histopathology because of the ease in visualizing cerebral damage by naked eyes without the need for microscopes or any other complicated instrumentation. In addition, quantitative analysis by colorimetric modality provided more precise evidence to evaluate ECM impairment. These results showed that increasing worm loads concomitant with low density of *P. berghei* resulted in higher protection against ECM. A previous study [29] established that INF-γ was required for the development of ECM during *P. berghei* infection in C57BL/6 mice. Here, INF-γ-producing CD4^+^ T cells, but not innate or CD8^+^ T cells promoted the development of ECM. The cerebral pathology during ECM is an IFN-γ-dependent process as evidenced by the complete resistance of the IFN-γ deficient mice to ECM [30]. IFN-γ is responsible for the induction of chemokines CXCL9 and CXCL10 during ECM [31], which subsequently recruit CD4^+^ and CD8^+^ T cells to the brain [32,33], with the latter one worked as the key role for pathogenicity. The current study also showed the expansion of splenic CD4^+^ and CD8^+^ T cells during the course of *P. berghei* infection although the phenotypes of T cells infiltration in the brain differed between the co-infected and *P. berghei*-mono-infected mice; CD8^+^ T cells were lower in co-infected mice when compared to *P. berghei-*mono-infected mice, and increasing the worm load during the co-infection further turned down the levels of CD8^+^ T cells. These results were consistent with the associated ECM survival data.

Among the various studies performed to explore the immunomodulatory mechanisms during malaria co-infection, most have been on infections with different *Schistosoma* species and various *Plasmodium* species*,* which is similar to the current study but thus far none had addressed the effect of parasite densities during co-infection. Waknine-Grinberg *et al.*[22] performed a study and showed that increased cercarial dose provided more protection against ECM, which is consistent with results from the current study. The regulatory responses and associated cytokines investigated in the current study offer more insights into the immune responses during such infections. This study clearly demonstrates that both the *Schistosoma* and *Plasmodium* densities play major roles in modulating the immune responses of the vertebrate host during co-infection. These findings offer useful insights to understand the complex array of immune responses to malaria co-infections in murine models that may explain why certain individuals develop CM while others exhibit only minor symptoms in human malaria infections.

## Conclusions

The results of this study demonstrate that changes in parasite density, both worm loads and *Plasmodium* parasite count, could modulate protection against ECM during the *S. japonicum-P. berghei*-co-infection. Increased cercariae co-infected with low density of *P. berghei* resulted in higher protection against ECM, while co-infection with higher density of *P. berghei* led to a converse result. Alterations in the regulatory response appear to play a key role in this adaptation.

## Abbreviations

BBB: Blood–brain-barrier; CM: Cerebral malaria; ECM: Experimental cerebral malaria; mAb: Mono-clone antibody; pRBCs: Parasitized red blood cells; RT: Room temperature; Tregs: Regulatory T cells.

## Competing interests

The authors declare that they have no competing interests.

## Authors’ contributions

All of the authors collaborated on the work presented in this study. M-lW, Y-mC and E-jL defined the research theme; M-lW prepared the infected animal models, designed the methods, performed parts of the experiments, and interpreted the results; WP, Y-hF and Z-mQ performed some of the experiments; YZ and Y-jG analysed the data; and M-lW, YZ, E-jL, and Y-mC drafted the manuscript. All authors read and approved the final version of the manuscript.
